# Special issue: avian malaria

**DOI:** 10.1017/S0031182024000040

**Published:** 2023-12

**Authors:** Lisa C. Ranford-Cartwright

**Affiliations:** School of Biodiversity, One Health and Veterinary Medicine, College of Medical, Veterinary and Life Sciences, University of Glasgow, Glasgow UK

**Keywords:** birds, *Haemoproteus*, Haemosporidia, *Leucocytozoon*, *Plasmodium*

## Abstract

Avian malaria parasites or haemosporidia are found in bird species worldwide. This special issue focuses on 3 most commonly studied genera: *Haemoproteus*, *Plasmodium* and *Leucocytozoon*. Seven research articles and reviews are provided to illustrate the breadth of knowledge of the diversity of avian malaria parasites in different regional habitats and across bird species, and the use of avian haemosporidian systems to examine host–parasite eco-evolutionary questions.

## Graphical abstract

**Figure:**
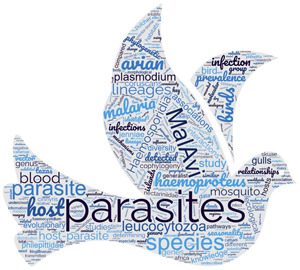

## Introduction

Haemosporidia are a large group of intracellular parasitic protozoa which infect amphibians, reptiles, birds and mammals, and which use haematophagous dipteran insects as vectors. This special issue focuses on Avian haemosporidia, often called Avian malaria parasites, which played important roles in early research on human malaria (Cox, 2010), but also are of major importance in their own right as parasites of bird species across the world (Valkiunas, 2005). Avian haemosporidia are important economically for their effects on productivity and mortality in domestic birds, and in captive birds in zoos and aviaries. In wild birds, they have been shown to impact negatively on the body condition, survival, migration and reproduction of their hosts (Lapointe *et al*., 2012). Avian haemosporidian parasites have also been useful as model systems to explore evolutionary and ecological parasite–host relationships (Dunn and Outlaw, 2019).

The different haemosporidian genera have different lifecycles in the avian host. Depending on the genus, asexual (mitotic) replication takes place in the reticuloendothelial cells of the tissues, and/or the red blood cells, where the parasites multiply as haploid clones. The sexual stages (gametocytes) of all genera are found in the circulating red blood cells, where they are taken up by the insect vectors during blood feeding, and the sexual phase is completed in the vector.

Avian haemosporidia are found on all continents except Antarctica, infecting a very large range of host species. There are more than 200 morphologically-distinct species of *Plasmodium*, *Haemoproteus* and *Leucocytozoon* that develop in a variety of bird and vector species. *Plasmodium* is often considered to be a generalist, transmitted between different avian orders, whereas *Leucocytozoon* and *Haemoproteus* species tend to exhibit a higher degree of specialism towards their vertebrate hosts (Valkiunas, 2005). The diversity of haemosporidia is closely linked to the richness of avian species, so the parasite community present in a specific location is influenced by the composition of avian host species, as well as by the distribution of vectors.

*Haemoproteus* are, globally, the most prevalent of the haemosporidia. Over 150 species have been defined, categorized into 2 subgenera: *Haemoproteus* comprises species that are transmitted by Hippoboscidae (louse flies), and *Parahaemoproteus* are species transmitted by Ceratopogonidae (biting midges). Over 40 species within the genus *Plasmodium* affect birds; the parasites are transmitted by mosquitoes, mainly Culicidae (e.g. *Culex*). *Plasmodium relictum* is one of the most widespread avian malaria parasites in the world, and has been linked to the extinction of some bird species (Atkinson and Samuel, 2010). *Leucocytozoon* parasites are unique amongst the avian haemosporidia as they only infect birds. The genus is the least studied of the avian haemosporidia. There are over 45 morphologically distinct species (Valkiunas and Iezhova, 2023). Leucocytozoa are transmitted by black flies (Simuliidae), except for 1 species, *Leucocytozoon caulleryi,* which is transmitted by biting midges (Ceratopogonidae).

The prevalence of avian haemosporidia was shown to be influenced by factors including temperature, altitude, latitude and season (reviewed in Sehgal, 2015). Generally, *Haemoproteus* is more prevalent at higher elevations and in more arid environments. *Leucocytozoon* is also found more commonly at higher elevations, whereas *Plasmodium* species are more frequently found at lower elevations. Prevalence has also been shown in some studies to be affected by the bird host age, sex, migratory status, foraging behaviour (whether at ground level or in the canopy), body mass and plumage colour (reviewed in Fecchio *et al*., 2021). Part of the explanation for these observations lies in the vector distribution and behaviour: blackflies which transmit *Leucocytozoon* species are more prevalent at higher altitudes, whereas mosquitoes which transmit *Plasmodium* are found in higher abundance at lower elevations. For *Haemoproteus* species, the Hippoboscidae vectors (louse flies) live most of their adult life on their host (obligate ectoparasitism), and therefore may be less influenced by different habitats in which their hosts move, whereas *Parahaemoproteus* vectors, biting midges (Ceratopogonidae), are opportunistic feeders on birds (free living ectoparasites).

The avian haemosporidia have very high genetic diversity. Studies on species diversity, host range and geographical distribution of avian haemosporidia were greatly aided by the development of a sequence database of *cytochrome b* gene lineages, known as MalAvi (Bensch *et al*., 2009). The database currently catalogues parasites from over 2000 bird species with around 5000 unique lineages in total, over 2000 of which are *Haemoproteus* species, and the remaining lineages are split evenly between *Leucocytozoon* and *Plasmodium* species.

## Scope of the special issue

The present special issue has 6 articles and 1 review. It begins with 3 papers describing the diversity of avian haemosporidia in 2 distinct habitats in Northern and Southern America respectively, and within larid seabirds in South Africa. The 4th paper describes a detailed phylogenetic analysis of a new species of *Leucocytozoon*, identified in a terrestrial bird in Brazil. The next 2 papers address evolutionary questions in avian malaria parasites, one examining host–parasite co-phylogeny in *Haemoproteus* infections in passerine birds in the United Kingdom, and the other haemosporidian infections in suboscine passerine birds, the Asities, native to Madagascar. Finally, the issue closes with a review of vector immune responses to infection with haemosporidian parasites. A brief summary of the individual contributions is given below.

**Viridiana Martinez** and colleagues report on the diversity of avian haemosporidia in the Davis Mountain sky islands of West Texas (Martinez *et al*., 2023). Sky islands are distinct, isolated mountain habitats, characterized by great species richness, surrounded by radically different lowland habitats such as deserts. The Davis Mountain region has rich species diversity with over 277 bird species, and also forms a temperate breeding ground for migrating birds. Both resident and migratory bird species were sampled, and more than 40% were found to be infected with haemosporidian parasites. *Haemoproteus* was the most common genus, but *Plasmodium* and *Leucocytozoon* were also present, and a small number of birds were infected with multiple species or lineages. The overall prevalence of infection, and the lineages present, were comparable to that seen in similar habitats in New Mexico, although 55% of the lineages found were previously unreported. Specialist lineages were more common amongst *Haemoproteus* than *Plasmodium* infections, which were mostly generalist. Comparing migratory and resident species, the authors concluded that host phylogeny played a more significant role in parasite prevalence than migratory status. Adult birds had higher prevalence than hatch-year birds, which the authors suggest could be due to a down-regulation of immune responses during reproduction, an increased exposure to vectors during nesting or foraging activities, or a higher mortality in infected juveniles.

**Daniela de Angeli Dutra** and co-authors studied how host phylogeny and seasonality influenced haemosporidian infection in the Caatinga, a seasonally dry tropical forest in Brazil, with over 200 bird species (de Angeli Dutra *et al*., 2023). Over 900 samples were taken over 4 seasons, and they found a higher diversity and prevalence of haemosporidian infections than has been reported from other regions of Brazil: an average prevalence of 51% with 32 different lineages. *Haemoproteus* was the most common genus, followed by *Plasmodium* (*Leucocytozoon* was not studied as it is believed to be at low prevalence in Brazil). Five lineages of *Haemoproteus* were found, mainly in columbiformes, with 2 lineages found in passerines. Seventeen lineages of *Plasmodium* were found, mainly in passerines. Parasite prevalence was found to be influenced by seasonality, with higher prevalence in the rainy season for *Plasmodium*, but in the dry season for *Haemoproteus*. This was perhaps surprising since the vectors are most abundant during the rainy season, but columbiformes were more common in the dry season. Overall, the study revealed a very high level of phylogenetic association between haemosporidian infection in birds: closely-related avian hosts harboured similar prevalence patterns within the community. In contrast with previous studies in Brazil which found *Plasmodium* to be the most common haemosporidian genus, the high prevalence of *Haemoproteus* observed could be explained by the high abundance of columbiformes in the Caatinga.

**Ralph Vanstreels** and co-authors present a survey of infections in Laridae aquatic birds – a suborder with more than 170 species including gulls, skuas and puffins (Vanstreels *et al*., 2023). Previous studies in this avian suborder revealed a low diversity of haemosporidian parasites, which could be explained by a limited sampling effort, or to their occupation of habitats with less abundant vectors. They reported 4 species of *Haemoproteus* in 2 gull species in South Africa. Almost 20% of wild kelp gulls (*Larus dominicanus*) were found to be infected with *Haemoproteus jenniae*, the first record of this species in Africa, and only the 4th previous recording globally, forming a new *cytb* lineage different to the existing *H. jenniae* lineages. This species was also found, for the first time, in 1 sample of a Hartlaub's gull (*Larus hartlaubii*), and thus appears to be distributed across Laridae seabirds. In addition, the authors provide a redescription of *Haemoproteus skuae* taken from a brown skua – previously described only once from the same host species, in the same location in Cape Town, in 2010.

Utilizing morphological and molecular data, **Lis Vieira** and co-authors report on the analysis of the first mitochondrial genome of a new species of *Leucocytozoon* found in a non-migratory Brazilian bird, the Red-legged Seriema (*Cariama cristata*), the first report of a competent host for leucocytozoa in Brazil (Vieira *et al*., 2023). The new species is named *Leucocytozoon cariamae*, and is distinguished from the *Leucocytozoon fringillarum* group by its microgamete morphology. The most closely-related species based on the mitochondrial genomes are leucocytozoa found in birds of different orders (tawny owls, American kestrel), but with different and distinct morphology. Leucocytozoon transmission has previously been deemed negligible outside the highland areas of Brazil, despite the presence of the vector blackflies, but the authors suggest that submicroscopic infections may be present in the Neotropics at low- and mid-elevations. The bird was also coinfected with *Haemoproteus pulcher*, and the authors present additional analyses of the mitochondrial genomes of *H. pulcher* and *Haemoproteus catharti* that suggest these species are more closely related to the reptile parasites *Haemocystidium,* with which they form a monophyletic group.

### Cospeciation, co-phylogeny and the origins of haemosporidia

As parasites and their hosts co-evolved, some parasites specialized to 1 host species, with high efficiency, whereas others adapted to several hosts, with a trade-off of lower efficiency. Specialization can trigger evolutionary arms races and can result in speciation in both parasite and host (co-speciation). Processes such as host-switching, where parasites successfully infect and adapt to a new host, duplication, where a parasite diverges and speciates within its original host, sorting, where a parasite becomes extinct within a host species, and inertia, where hosts speciate but parasites fail to diverge, all affect host–parasite relationships.

**Charlie Woodrow** and co-authors have used *Haemoproteus* infections in passeriform bird hosts to investigate host–parasite co-phylogeny (Woodrow *et al*., 2023). Sampling 32 passerine species in the south of England, they found almost 60% to be infected with *Haemoproteus*, and identified 30 lineages, 9 of which were novel (i.e. not in the MalAvi database). 23 lineages were found only in 1 bird species. The highest parasite richness was found in the blackcap *Sylvia atricapilla*, which also had the highest parasite specificity – 7 lineages were found, 6 of which were exclusive to the blackcap. The presence of a generalist parasite lineage masked an underlying co-phylogeny – removing this from the analysis allowed the identification of host-switching and duplication as contributory factors favouring coevolution of parasite and host.

**Hannah Barbon** and co-authors present a study of haemosporidian infections in a family of birds – the Asities (Philepittidae) – that are native to Madagascar (Barbon *et al*., 2023). The Asities represent a single radiation, with 4 extant species in 2 genera, that feed on fruit (*Philepitta* spp.) and nectar (*Neodrepanis* spp.). They compared parasite infection in *Neodrepanis* spp. to that found in Malagasy sunbirds (*Cinnyris* spp.), with which they share habitat and nectar foraging behaviour, to test if ecological factors were stronger influences on parasite prevalence than phylogenetic relationships between the avian hosts. They found a high prevalence of haemosporidian infection in all bird taxa studied. *Cinnyris* species were found to have predominantly specialist haemosporidian lineages, whereas Philepittidae were found to have mainly generalist lineages, with no *Haemoproteus* and few mixed infections, suggesting that these birds may be resistant to the parasite species circulating in Madagascar. Haemosporidian parasite composition was more similar for more closely related avian hosts than those that shared habitat and foraging behaviour, and the authors conclude that haemosporidian infections are driven primarily by host phylogenetic rather than ecological factors.

### Vectors

Finally, **Irene Hernandez-Caballero** and colleagues present a systematic review of vector responses to infection with haemosporidian parasites, focussing on gene expression in *Culex quinquefasciatus* mosquitoes during infection with *P. relictum*, and comparing the results with *Anopheles* infection with human malaria *Plasmodium falciparum* (Hernandez-Caballero *et al*., 2023). They discuss the relative importance of the 3 main immune response pathways: Toll, Imd and JAK/STAT, as well as changes in metabolic pathways observed during infection in the different parasite–vector combinations. The authors also highlight the relative paucity of studies on avian malaria – vector interactions.

## Summary

The papers presented in this special issue serve to highlight the extreme diversity within the avian malaria parasites, and the growing use of these parasites to explore evolutionary questions. Avian hosts globally exhibit a high prevalence of infection with haemosporidian parasites. Both generalist and specialist lineages of haemosporidians are common, and all papers report novel lineages, adding to the thousands already in the MalAvi database. Host phylogeny is repeatedly identified in the papers presented here as significant in explaining lineage prevalence. The description of a new species, *Haemoproteus cariamae*, and the redescription of *H. skuae* continue the long history of **Parasitology** in the taxonomy of parasite species.

## References

[ref1] Atkinson CT and Samuel MD (2010) Avian malaria *Plasmodium relictum* in native Hawaiian forest birds: epizootiology and demographic impacts on `apapane *Himatione sanguinea*. Journal of Avian Biology 41, 357–366.

[ref2] Barbon H, Berthoud JL, Woog F and Musa S (2023) Haemosporidian parasite infections of Malagasy Philepittidae and Nectariniidae are driven by phylogeny rather than ecology. Parasitology 150, 1316–1329. 38087861 10.1017/S0031182023001075 PMC10941219

[ref3] Bensch S, Hellgren O and Perez-Tris J (2009) MalAvi: a public database of malaria parasites and related haemosporidians in avian hosts based on mitochondrial cytochrome b lineages. Molecular Ecology Resources 9, 1353–1358. 21564906 10.1111/j.1755-0998.2009.02692.x

[ref4] Cox FEG (2010) History of the discovery of the malaria parasites and their vectors. Parasites & Vectors 3, 5. 20205846 10.1186/1756-3305-3-5 PMC2825508

[ref5] de Angeli Dutra D, Khan AU, Ferreira FC, Beirão MV, Pichorim M, Moreira PA and Braga ÉM (2023) Host phylogeny and seasonality shapes avian haemosporidian prevalence in a Brazilian biodiverse and dry forest: the Caatinga. Parasitology 150, 1277–1285. 37246557 10.1017/S0031182023000549 PMC10941212

[ref6] Dunn JC and Outlaw DC (2019) Flying into the future: avian haemosporidians and the advancement of understanding host–parasite systems. Parasitology 146, 1487–1489. 31084650 10.1017/S003118201900057X

[ref7] Fecchio A, Clark NJ, Bell JA, Skeen HR, Lutz HL, De La Torre GM, Vaughan JA, Tkach VV, Schunck F, Ferreira FC, Braga ÉM, Lugarini C, Wamiti W, Dispoto JH, Galen SC, Kirchgatter K, Sagario MC, Cueto VR, González-Acuña D, Inumaru M, Sato Y, Schumm YR, Quillfeldt P, Pellegrino I, Dharmarajan G, Gupta P, Robin VV, Ciloglu A, Yildirim A, Huang X, Chapa-Vargas L, Álvarez-Mendizábal P, Santiago-Alarcon D, Drovetski SV, Hellgren O, Voelker G, Ricklefs RE, Hackett SJ, Collins MD, Weckstein JD and Wells K (2021) Global drivers of avian haemosporidian infections vary across zoogeographical regions. Global Ecology and Biogeography 30, 2393–2406.

[ref8] Hernandez-Caballero I, Hellgren O and Garcia-Longoria L (2023) Genomic advances in the study of the mosquito vector during avian malaria infection. Parasitology 150, 1330–1339. 37614176 10.1017/S0031182023000756 PMC10941221

[ref9] Lapointe DA, Atkinson CT and Samuel MD (2012) Ecology and conservation biology of avian malaria. Annals of the New York Academy of Sciences 1249, 211–226. 22320256 10.1111/j.1749-6632.2011.06431.x

[ref10] Martinez V, Keith KD, Grace JK and Voelker G (2023) Avian haemosporidians of breeding birds in the Davis Mountains sky-islands of west Texas. Parasitology 150, 1266–1276. 38072659 10.1017/S0031182023001087 PMC10941211

[ref11] Sehgal RNM (2015) Manifold habitat effects on the prevalence and diversity of avian blood parasites. International Journal for Parasitology: Parasites and Wildlife 4, 421–430. 26835250 10.1016/j.ijppaw.2015.09.001 PMC4699977

[ref12] Valkiunas G (2005) Avian Malaria Parasites and Other Haemosporidia. Boca Raton, Florida: CRC Press.

[ref13] Valkiunas G and Iezhova TA (2023) Insights into the biology of leucocytozoon species (Haemosporida, Leucocytozoidae): why is there slow research progress on agents of leucocytozoonosis? Microorganisms 11, 1251. 10.3390/microorganisms11051251 PMC10221462 37317225

[ref14] Vanstreels RET, Chagas CRF, Valkiūnas G, dos Anjos CC, Parsons NJ, Roberts DG, Snyman A, Hurtado R, Kirchgatter K, Ludynia K and Pistorius PA (2023) *Haemoproteus jenniae* (Haemoproteidae, Haemosporida) infects gulls (*Larus* spp.) in South Africa, with redescription of *Haemoproteus skuae*. Parasitology 150, 1286–1295. 36951108 10.1017/S003118202300029X PMC10941229

[ref15] Vieira LMC, Pereira PHO, Vilela DAR, Landau I, Pacheco MA, Escalante AA, Ferreira FC and Braga ÉM (2023) *Leucocytozoon cariamae* n. sp. and *Haemoproteus pulcher* coinfection in *Cariama cristata* (Aves: Cariamiformes): first mitochondrial genome analysis and morphological description of a leucocytozoid in Brazil. Parasitology 150, 1296–1306. 37655743 10.1017/S0031182023000811 PMC10941214

[ref16] Woodrow C, Rosca AT, Fletcher RM, Hone AL, Ruta M, Hamer KC and Dunn JC (2023) *Haemoproteus* parasites and passerines: the effect of local generalists on inferences of host–parasite co-phylogeny in the British Isles. Parasitology 150, 1307–1315. 37395052 10.1017/S0031182023000628 PMC10941225

